# Scaling diffraction data in the *DIALS* software package: algorithms and new approaches for multi-crystal scaling

**DOI:** 10.1107/S2059798320003198

**Published:** 2020-03-31

**Authors:** James Beilsten-Edmands, Graeme Winter, Richard Gildea, James Parkhurst, David Waterman, Gwyndaf Evans

**Affiliations:** a Diamond Light Source Ltd, Harwell Science and Innovation Campus, Didcot OX11 0DE, United Kingdom; b STFC, Rutherford Appleton Laboratory, Didcot OX11 0FA, United Kingdom; c CCP4, Research Complex at Harwell, Rutherford Appleton Laboratory, Didcot OX11 0FA, United Kingdom

**Keywords:** diffraction, crystallography, multi-crystal, data analysis, scaling

## Abstract

In processing X-ray diffraction data, the intensities obtained from integration of the diffraction images must be corrected for experimental effects in order to place all intensities on a common scale both within and between data collections. Scaling corrects for effects such as changes in sample illumination, absorption and, to some extent, global radiation damage that cause the measured intensities of symmetry-equivalent observations to differ throughout a data set. This necessarily requires a prior evaluation of the point-group symmetry of the crystal. This paper describes and evaluates the scaling algorithms implemented within the *DIALS* data-processing package and demonstrates the effectiveness and key features of the implementation on example macromolecular crystallographic rotation data. In particular, the scaling algorithms enable new workflows for the scaling of multi-crystal or multi-sweep data sets, providing the analysis required to support current trends towards collecting data from ever-smaller samples. In addition, the implementation of a free-set validation method is discussed, which allows the quantification of the suitability of scaling-model and algorithm choices.

## Abstract 2

A new scaling program is presented with new features to support multi-sweep workflows and analysis within the *DIALS* software package.

## 1. Introduction   

The first major step in processing crystallographic data is the integration of diffraction images, *i.e.* the extraction of a set of intensities and error estimates from the raw data. These integrated intensities are a product of *F* ^2^, the squares of the structure-factor amplitudes, and experimental effects: the aim of scaling is to correct for the experimental contributions to the measured intensities to give a set of intensities proportional to *F* ^2^. Scaling will typically account for systematic effects such as changes in sample-illumination volume, beam intensity, secondary beam absorption and global radiation damage during a single-sweep measurement, which are multiplicative in their effect on the measured intensities. The scaling process also places the intensities from multiple sweeps on a common scale. Once scales have been applied to all reflections, symmetry-equivalent reflections can be merged together. Several programs exist to scale macromolecular diffraction data, including *SCALA* (Evans, 2006 ▸), *AIMLESS* (Evans & Murshudov, 2013 ▸), *SADABS* (Sheldrick, 1996 ▸), *SCALEPACK* (Otwinowski & Minor, 1997 ▸) and *XDS*/*XSCALE* (Kabsch, 2010*b* ▸).

In recent years, technical advancements at synchrotron X-ray sources, including the introduction of high-frame-rate pixel-array detectors, have spurred changes in data-collection methodologies, including an increasing focus on serial approaches to crystallography (Stellato *et al.*, 2014 ▸; Grimes *et al.*, 2018 ▸). Increasingly brighter sources allow useful data collections from microcrystals, but require different data-collection approaches owing to shorter crystal lifetimes and the practicalities of efficiently mounting and measuring numerous microcrystals, such as *in situ* data collection, as at the VMXi (Sanchez-Weatherby *et al.*, 2019 ▸) and I24 (Axford *et al.*, 2012 ▸) beamlines at Diamond Light Source. These developments have spawned new approaches to data collection, such as multi-sweep stepped transmission collection (Winter *et al.*, 2019 ▸), and place an increasing burden on the data-analysis step. These advancements necessitate improved algorithms and workflows for processing multi-sweep data sets, particularly in the scaling and merging step, where the whole data set must be analysed as one.

To provide new tools and algorithms for data analysis of novel approaches to diffraction data collection, the *DIALS* (Winter *et al.*, 2018 ▸) software package has been developed, with an initial focus on the integration of data from pixel-array detectors. This paper reviews current methods for scaling and symmetry determination before describing and evaluating the implementation of scaling algorithms within *DIALS*, demonstrating use cases by analysing a number of example macromolecular data sets. This work builds upon the strengths of previous approaches, including the implementation of two types of scaling models currently used in existing scaling programs, with the core algorithm incorporating aspects of the approaches taken by the programs *AIMLESS* (Evans & Murshudov, 2013 ▸) and *XDS*/*XSCALE* (Kabsch, 2010*b* ▸). In addition, a number of workflows have been developed to facilitate the scaling of multi-sweep data sets, and a free-set validation approach has been implemented to assess the suitability of a given scaling model by providing an assessment of overfitting. This work therefore extends the scope of *DIALS*, enabling the processing of diffraction data from raw images to scaled intensities suitable for structure solution.

## 2. Background   

### 2.1. Formalism of scaling   

Following the formalism of Hamilton *et al.* (1965 ▸), an inverse scale factor *g_hl_* is determined for each reflection and then applied in order to correct the data, *i.e.*

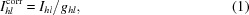
where *I_hl_* is the *l*th observation of symmetry-unique reflection *h*. The factors *g_hl_* are determined by minimizing the function 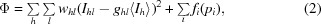
where 〈*I_h_*〉 is the current best estimate of the true intensity of symmetry-unique reflection *h*, *w_hl_* are weighting factors and the second term is a general restraint term for the scaling-model parameters *p_i_*. The best least-squares estimate of 〈*I_h_*〉 is derived from the data by minimizing Φ with respect to 〈*I_h_*〉, giving 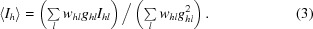
The best least-squares estimates of the inverse scale factors are found using weighting factors that are proportional to the inverse variances of the intensities. To determine the inverse scale factors, one must create a parameterized scaling model from which the scale factors are calculated: in this implementation, the model is determined by minimizing the least-squares target function with respect to the model parameters.

### 2.2. Symmetry determination prior to scaling   

Prior to scaling, the point-group symmetry of the data set must be known, as the intensities are grouped by symmetry-unique index during scaling. It is possible to scale in all point groups lower than the true point group of the crystal; however, this would not account for the equivalence of reflections related by the absent symmetry operations. Several programs exist to perform symmetry analysis, including *POINTLESS* (Evans, 2006 ▸) and *XDS* (Kabsch, 2010*b* ▸). In *POINTLESS*, each symmetry element of the lattice is scored by comparing the intensities of reflections related by symmetry, and the combination of different symmetry elements and their scores provides a score for a given Laue group; systematic absences can also be assessed to score potential space groups. In *XDS*, *R*_meas_ is calculated for all enantiomorphous groups consistent with the lattice, and the highest symmetry group with an acceptable *R*_meas_ is chosen (Kabsch, 2010*a* ▸).

An analysis of the point-group symmetry must also account for indexing ambiguities in polar point groups in the case of multiple sweeps, or accidental ambiguity for sparse multi-crystal data sets (Brehm & Diederichs, 2014 ▸; Gildea & Winter, 2018 ▸). For wide-rotation data sets, indexing ambiguities can be resolved by reindexing against a reference data set. For sparse data sets, a different approach is required based on the assessment of correlation coefficients between data sets. Techniques include dimensionality reduction to separate clusters based on indexing choice (Brehm & Diederichs, 2014 ▸; Diederichs, 2017 ▸; Gildea & Winter, 2018 ▸) and the selective breeding method (Kabsch, 2014 ▸).

Within the *DIALS* package, symmetry-determination algorithms have been implemented in two programs. *dials.symmetry*, which will be described elsewhere, determines the space-group symmetry for single-sweep rotation data using algorithms similar to *POINTLESS* (indexing ambiguities between sweeps can be resolved by reindexing against a reference data set with *dials.reindex*). The second program, *dials.cosym*, implements multi-crystal symmetry determination as described in Gildea & Winter (2018 ▸), which includes the resolution of indexing ambiguities.

### 2.3. Scaling-model parameterization   

Many model parameterizations are possible; currently, there are two main approaches that are used for the scaling of macromolecular diffraction data. The first uses a multicomponent parameterization based on physical descriptors of the instrument, experiment and sample characteristics such as X-ray absorption by the sample, variations in illuminated volume and beam intensity, and global radiation damage. Such an approach is taken in *SCALA* (Evans, 2006 ▸), *AIMLESS* (Evans & Murshudov, 2013 ▸) and *SADABS* (Sheldrick, 1996 ▸). For example, *AIMLESS* uses a three-component model to account for scale, radiation damage and absorption effects. The scale and radiation-damage terms are parameterized as a smoothly varying function of rotation or time, whilst spherical harmonics are used to parameterize an absorption correction surface which rotates with the crystal. A similar model is used in *SADABS*, and both programs include the optimization of standard error estimates. The second approach to scaling-model parameterization is taken by *XDS*/*XSCALE* (Kabsch, 2010*b* ▸), in which the corrections are determined by sampling up to three two-dimensional arrays in succession, with the purpose of removing correlation of the measured intensities with image number, resolution and measured position on the detector. Both approaches to scaling-model parameterization have proved to be effective for correcting experimental data.

## 3. Implementation   

This section describes the implementation details of the key components of the scaling algorithms and potential scaling workflows.

### 3.1. Scaling-model parameterization   

Currently, three scaling models are defined within the *DIALS* package: the *physical* model, the *KB* model and the *array* model. The *DIALS* package is structured so that additional scaling models can be implemented by a user; further details can be found in the package documentation. For all models, a subset of possible components may be used, while the components can be refined sequentially or (by default) concurrently.

#### 3.1.1. The *physical* scaling model   

The default parameterization of the scaling model used in *DIALS* (referred to as the *physical* model) is based on that of *AIMLESS* (Evans & Murshudov, 2013 ▸), *i.e.* the model is composed of three components: a scale term *C_hl_*, a decay term *T_hl_* and an absorption term *S_hl_*, 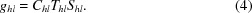
The scale term is intended to correct for the majority of intensity variation as a function of rotation, such as changes in illumination volume or average beam absorption. The decay term uses a relative global temperature factor (*B* factor) to account for changes in intensity as a function of resolution (*d*-spacing) to model changes in the bulk disorder of the crystal, *i.e.* global radiation damage (Garman, 2010 ▸). The absorption term accounts for relative variations in absorption for different scattering vectors in the crystal reference frame.

**Figure 1:**
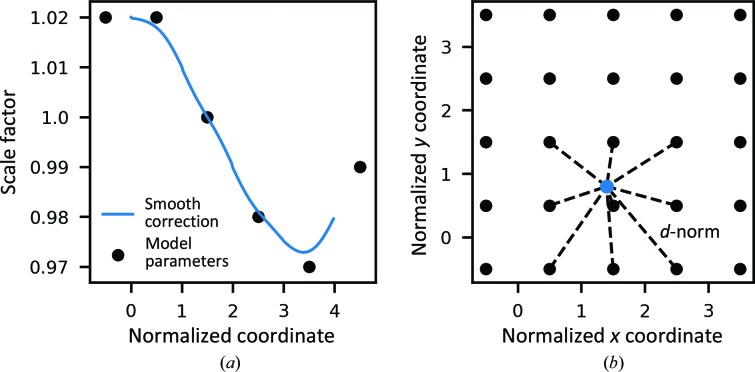
(*a*) Example of a 1D smooth scaling component. The scale factor at adjusted coordinate *x* is determined by a Gaussian-weighted average of the nearest three parameters at *x_i_*, with the weighting depending on the distances |*x* − *x_i_*|. (*b*) Generalization of smooth scaling in higher dimensions, shown in 2D. The value of the component at adjusted position (*x*, *y*) is a Gaussian-weighted average of the nearest three parameters along each dimension, with the weighting depending on the distances *d*-norm = [(*x* − *x_i_*)^2^ + (*y* − *y_i_*)^2^]^1/2^.

For the scale and decay terms, the correction is parameterized as a smooth function of rotation angle φ. Two adjusted angles are calculated, *r* = φ/α_1_ and *t* = φ/α_2_, which in general have different normalization factors (α_1_, α_2_), with *t* acting as a proxy for time from the start of the measurement (the assumption is of a constant rotation per frame within the sweep). The smoothing function consists of a Gaussian weighting over a set of regularly spaced parameters, *C_i_* and *B_i_*, spanning at least the respective ranges of adjusted angles. The correction factor at a given adjusted position is calculated using a Gaussian-weighted sum of the nearest *i* values (typically *i* = 3),
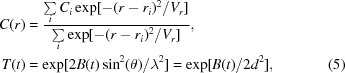
where 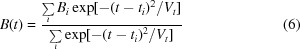
and *V* is a ‘variance’ to control the extent of smoothing. A schematic of a 1D smooth scaling component is shown in Fig. 1 ▸(*a*). A weak restraint term (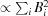) is used as part of the minimization target (2) to weakly restrain the relative *B* values towards zero. Typically, within *DIALS*, the parameter spacing is automatically determined to give sufficient sampling across the rotation range; parameters are separated by 10–20° of rotation for a full rotation sweep and 1–3° for narrow sweeps.

For the absorption term, a smoothly varying correction as a function of incoming beam and scattering vectors is applied by defining an absorption correction surface. Following the form of the absorption anisotropy term in Blessing (1995 ▸), spherical harmonics are used as a normalized 3D basis set for the correction surface, which is defined as a sum of spherical harmonic terms *Y_lm_*, 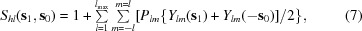
where the *P_lm_* prefactors are the model parameters to be minimized and −**s**_0_ and **s**_1_ are the reverse incident beam and scattering vectors in the crystal frame. A restraint term (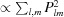) is used as part of the minimization target (2) to restrain the parameters towards zero, *i.e.* the absorption correction is restrained towards 1. As more harmonic terms are added to the sum in (7), the absorption correction surface is able to exhibit sharper features, enabling better modelling of the absorption variation for crystals with well defined corners/edges. As discussed in Evans (2006 ▸), including the odd harmonic terms can help to account for effects such as crystal miscentering, and a default *l*_max_ value of 4 is sufficient to give a good fit.

For a 360° sweep, the physical model therefore uses 70 parameters, comprising 26, 20 and 24 parameters for the scale, decay and absorption corrections, respectively.

#### 3.1.2. The *KB* scaling model   

A second model is provided, which can be considered to be a special case of the *physical* model and is referred to as the *KB* model. Here, a single scaling component *k* and *B* factor is applied to all reflections in the data set, 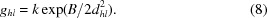
This model is suitable for still-shot images and may be preferable to the physical model for very narrow sweeps, where *C*(*r*) and *T*(*t*) are approximately constant and there is insufficient information to determine an absorption correction surface.

#### 3.1.3. The *array* scaling model   

A third model is also provided, following a more generalized approach to parameterization, motivated by that taken in *XDS*/*XSCALE* (Kabsch, 2010*b* ▸), and is referred to as the *array* model. This approach attempts to remove the correlation of measured intensities with rotation angle and measured position on the detector. The implementation of this approach within *DIALS* uses the product of up to three components, referred to as decay (*D_hl_*), absorption (*A_hl_*) and modulation (*M_hl_*) terms.

The decay term is parameterized by a 2D grid of parameters regularly spaced in adjusted rotation angle *r* and normalized ‘resolution’ *d*′ ∝ *d* ^−2^ (see Fig. 1 ▸ *b*). In a similar manner to the smooth scale and decay terms of the *physical* model, the scale factor for each reflection is given by a Gaussian-weighted average of the nearest *i*, *j* parameters in each dimension of a 2D array of parameters *C_ij_*, 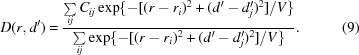
For the modulation term, a 2D array is defined over normalized detector pixel positions and smoothed values are obtained analogously, whereas for the absorption term a 3D array is defined over normalized detector pixel positions *x*′ and *y*′ and adjusted rotation angle, and an analogous 3D smoothing is used to determine values for *A_hl_* at a given *x*′, *y*′, *r*.

The default configuration of the array model in *DIALS* uses the decay and absorption corrections, with no modulation correction, as a detector-based correction at the scaling step is not always necessary for modern single photon-counting detectors. Excluding the modulation correction also reduces the correlation between scaling-model parameters and aids the stability of scaling in *DIALS*. This configuration enables corrections to be determined based on resolution, rotation angle and scattering vector (through the *x* and *y* dependence of the absorption correction), with the only assumption on the form of the scale correction being that it is smoothly varying in nature. For a 360° sweep, the array model therefore uses 240 parameters for the decay correction and 500 parameters for the absorption correction.

### 3.2. Selection of reflections for model optimization   

Owing to the small number of scaling-model parameters relative to the number of observations, scaling-model optimization is typically overdetermined and therefore a subset of the data can be used for computational efficiency when minimizing the scaling models; this may be critical for very large data sets (*e.g.* greater than a million observations) to reduce memory requirements and total runtime.

For scaling a single sweep, a random subset of the groups of symmetry-equivalent reflections are used for scaling-model optimization. For multiple sweeps, a random selection can also be used; however, it is necessary to ensure that a sufficiently connected subset of the data is selected for a stable minimization, *i.e.* low parameter uncertainty, particularly for the case of narrow-sweep multi-crystal data sets. To ensure this, in addition to a purely random selection on groups of symmetry equivalents, a ‘quasi-random’ inter-sweep reflection-selection algorithm was implemented. We define a quasi-random algorithm as an algorithm that selects a distribution similar to a random algorithm, in terms of the number of reflections per data set, but in a way that ensures higher connectivity than a random algorithm. To achieve this, the quasi-random algorithm builds a subset by successively choosing the symmetry group which spans the highest number of sweeps and that also has a reflection in the currently least-populated sweep in the subset. The subset is built in this way until at least a given number of reflections, or the maximum available reflections, have been selected for each sweep, thus ensuring that inter-sweep connected reflections are selected for all data sets. The algorithm is run on each resolution bin separately to give an inter-sweep connected subset which contains approximately the same number of reflections per data set, which are distributed approximately evenly in resolution. This is augmented by further random selections of groups for each sweep individually, and across sweeps, to form the subset of reflections to be used for scaling-model optimization.

For both single- and multi-sweep scaling, the number of reflections selected is controlled by two thresholds: the greater of a minimum number of groups and a minimum number of total reflections. The effect of these parameter values and the multi-sweep selection algorithm will be assessed in Section 4.4.

### 3.3. Adjustment of intensity uncertainties   

At the point of scaling, one can compare the expected and observed distribution of intensities and uncertainties (sigmas) to evaluate the suitability of the intensity uncertainties estimated during integration. It is recognized that these uncertainties typically underestimate the true errors in the data, as there are systematic experimental effects that will not have been fully accounted for (Evans, 2006 ▸; Diederichs, 2010 ▸). To correct for this, current scaling programs tend to use a variation of a two-parameter error model of the general form 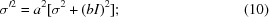
however, a third parameter giving a correction proportional to *I* is used by *AIMLESS* (Evans & Murshudov, 2013 ▸). In *DIALS*, a two-parameter error model is used to adjust the uncertainties, as this form has a simple physical interpretation: the *b* parameter models systematic errors that scale with the intensity, whereas the *a* factor accounts for general systematic errors affecting the whole data set. These parameters can then be related to the asymptotic *I*/σ limit (ISa; Diederichs, 2010 ▸) as 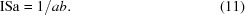
For an experiment with low systematic error, the *a* value is expected to be close to 1, with a *b* value around the range of 0.02–0.04.

To optimize the error model, a number of approaches are possible. One possibility is to use a linear regression by equating σ′^2^ with the observed variance of symmetry equivalents; however, outliers must be treated carefully. An alternative approach is an optimization based on analysis of the normalized intensity deviations, defined as (Evans & Murshudov, 2013 ▸) 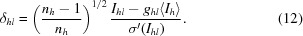
The assumption is that δ_*hl*_ should be normally distributed with a standard deviation of 1 across the intensity range measured. Within *DIALS*, the *a* parameter is estimated by using a linear regression to fit the central section of the normal probability plot (Howell & Smith, 1992 ▸); however, this requires an estimate of the *b* parameter. To determine an estimate for the *b* parameter, a minimization is performed to correct the standard deviation of δ_*hl*_ to 1.0 for the data binned by intensity (a principle described in Evans & Murshudov, 2013 ▸), which depends on the *a* estimate. These two methods are used in *DIALS* to sequentially estimate *a* and *b* until the respective values converge, starting with *a* = 1.0, *b* = 0.02. During the parameter estimation, the inverse-variance weights used in the estimation of 〈*I_h_*〉 are fixed to be the best estimate at the start of the error-model refinement, *i.e.* either the initial variances from integration or updated variances based on a previous round of error-model refinement. Further details of the implementation within *DIALS* are given in Appendix *A* [App appa]. Importantly, the fact that *a* and *b* are estimated separately removes the need for restraints during the analysis of intensity dependence, while using aggregate data properties such as the standard deviation of δ_*hl*_ and the central part of the normal probability plot reduces the influence of data with high deviations, giving error-model parameters that suitably describe the data *on average*.

### 3.4. Scaling algorithms and workflows for multi-data-set scaling   

**Figure 2:**
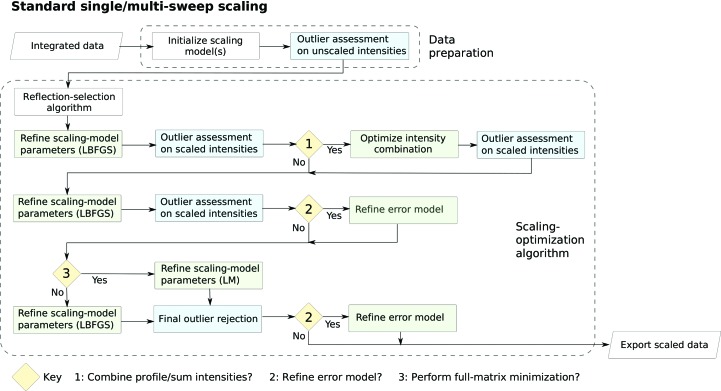
Flow chart showing the main processes of the default scaling algorithm, which consists of several rounds of model optimization and outlier rejection, as well as optimization of profile and summation intensity combination and adjustment of uncertainty (error) estimates by refining a two-parameter error model.

The principal steps of the single-sweep scaling algorithm used in *DIALS* are shown in Fig. 2 ▸. Parts of the algorithm that can be disabled by the user are indicated by the decision nodes; however, by default all steps are performed.

At each step of scaling-model refinement, the minimization is performed using the initially determined subset of reflections minus identified outliers. The minimization is performed using the *lstbx* subpackage (Bourhis *et al.*, 2015 ▸) of the *Computational Crystallography Toolbox* (*cctbx*; Grosse-Kunstleve *et al.*, 2002 ▸). Except for the final minimization cycle, the minimization is performed using a quasi-Newton minimizer (L-BFGS method; Liu & Nocedal, 1989 ▸) to minimize the nonlinear equation (2). The advantage of this method is that it does not require the calculation of the Jacobian matrix, which can be computationally slow and memory-intensive for problems involving a large number of parameters or observations. The final minimization cycle uses a Levenberg–Marquardt full-matrix minimizer (Moré, 1978 ▸), which enables the model-parameter uncertainties to be determined via matrix inversion. These parameter uncertainties are propagated to give an estimation of the uncertainties of the scale factors for all reflections and an adjustment to the variance of the scaled intensities.

Several rounds of outlier assessment are performed during the scaling algorithm to retest all observations based on the updated models (even if they were previously determined to be outliers). Following the methods described in Evans (2006 ▸), an observation is classed as an outlier if its normalized deviation from the weighted mean of the group of symmetry equivalents, excluding the test reflection, is above a given threshold, which is 6σ by default. In the final round of outlier assessment, reflections determined as outliers are marked as such for output and merging.

Another important consideration during scaling is whether the profile or summation intensity estimates give the most reliable estimates of the measured intensity. In general, profile fitting improves the estimation of weak intensities by reducing the random counting error; however, for very large intensities the error in the profile model can be greater than the counting error (Leslie, 1999 ▸). After the first cycle of scaling and outlier rejection, which uses profile intensity estimates, the optimal intensity choice is investigated. To reflect the fact that summation estimates can be better than profile estimates for very large intensities, a smooth crossover from profile to summation intensities, as a function of uncorrected intensity, can be defined (Evans & Murshudov, 2013 ▸): 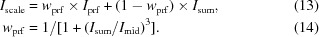
At the ‘optimize intensity combination’ step, a number of logarithmically spaced crossover *I*_mid_ values are tested, and the choice (summation/profile) or combination with the lowest *R*_meas_ is used for the remainder of scaling and output. Here, *R*_meas_ is favoured over CC_1/2_, as the CC_1/2_ values are typically all very similar to unity, hindering discernment of the optimal choice.

The final key component is the optimization of the error model to give an improved estimate of the intensity uncertainties. After the first instance of error-model optimization, the improved error estimates are used to update the weights/uncertainties in subsequent rounds of scaling-model optimization and outlier rejection. For the penultimate step, the error model is minimized again and the variances of all reflections are updated before data output. At the point of output, the data can be merged using algorithms available in *cctbx* or retained in unmerged format. The merging process has also been packaged into the command-line program *dials.merge*, which produces a merged MTZ file containing intensities and ‘truncated’ amplitudes suitable for structure solution (French & Wilson, 1978 ▸).

**Figure 3:**
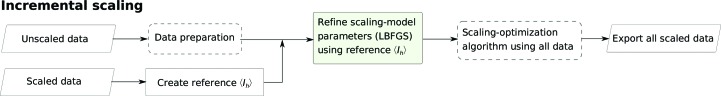
Flow chart showing the stages of the incremental scaling algorithm, in which an additional prescaling round is performed using the already scaled data as a reference data set.

For scaling multiple sweeps, one possibility is to scale all sweeps concurrently: if none of the sweeps have previously been scaled, the first round of outlier rejection is performed on the individual sweeps before all sweeps are combined for the scaling-optimization algorithm (Fig. 2 ▸). A novel feature in *DIALS* is the support for a workflow in which individual sweeps can be added one or more at a time to build up the whole data set incrementally. This is supported by a round of scaling against a reference, shown in Fig. 3 ▸, where the unscaled sweeps are scaled against reference 〈*I_h_*〉 values calculated from previously scaled data. This acts to quickly scale the new sweep based on the current best estimates of 〈*I_h_*〉, before the data are combined and passed to the scaling-optimization algorithm, to further minimize the scaling models for all sweeps. This process can be repeated for additional sweeps, allowing the full data set to be built up incrementally. One advantage of this method is that as the data set is built the estimates of 〈*I_h_*〉 become closer to the true 〈*I_h_*〉, so that when a new sweep is added the majority of its model optimization can be performed in the quick reference scaling before all scaling models are adjusted to account for the combined 〈*I_h_*〉 estimate. Additionally, the incremental workflow allows inspection and evaluation of the scaled data set after each addition, enabling incorporation into data-collection and processing routines, providing essential, near-real-time feedback to users of synchrotron beamline facilities.

**Figure 4:**
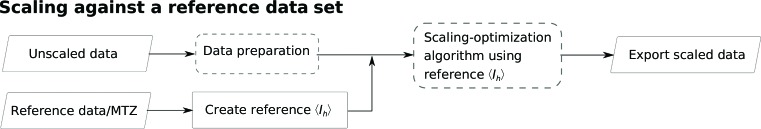
Flow chart showing the stages of the reference scaling algorithm, which uses the scaling-optimization algorithm with the reference set of intensities in the minimization target.

The algorithms in *DIALS* also allow scaling against a reference data set (Fig. 4 ▸). Practically, a merged MTZ file or a *DIALS* reflection file can be used to give input intensities with which to calculate the reference 〈*I_h_*〉. The scaling-optimization algorithm is then run only on the nonreference data using the reference 〈*I_h_*〉, with only the nonreference data exported. An example use case for these modes includes time-resolved pump–probe experiments, where one may have a full measurement of a test crystal as a reference followed by short time-resolved measurements of many crystals, which should all be scaled against the reference crystal, placing the time-resolved data on a common scale.

### 3.5. Data-set selection for multi-crystal scaling   

To process multi-crystal narrow-sweep data sets, where non-isomorphism and radiation damage can be significant, it is necessary to consider the suitability of all data sets for scaling and merging before accepting the best combined scaled data set.

One approach to data-set selection is to perform hierarchical clustering based on unit-cell parameters, as performed in the program *BLEND* (Foadi *et al.*, 2013 ▸), where clustering is performed before scaling. Another approach, described in Giordano *et al.* (2012 ▸), is to perform hierarchical clustering based on the correlation-coefficient matrix between data sets after scaling. A further approach based on optimizing the overall CC_1/2_ has been described by Assmann *et al.* (2016 ▸). It was shown that an approach that optimizes CC_1/2_, referred to as the ΔCC_1/2_ method, is favourable as a means to predominantly exclude data sets that are systematically different from the majority of the data set without a bias to exclude weak data sets. The contribution of a subset of the data to the overall CC_1/2_ is determined by calculating the ΔCC_1/2_ for the *i*th subset of the data, defined as 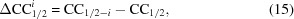
where CC_1/2−*i*_ is the overall CC_1/2_ excluding the *i*th subset. The σ–τ method (Assmann *et al.*, 2016 ▸) is used to reliably calculate CC_1/2_ in resolution bins, while a single CC_1/2_ value per subset is given by the weighted average over the resolution bins. Subsets of the data with a highly negative ΔCC^*i*^_1/2_ can be considered to be significantly degrading the quality of the whole data set owing to systematic differences in the intensities.

For scaling multi-crystal data sets in *DIALS*, an option is available to perform successive cycles of scaling and exclusion, based on ΔCC_1/2_ values, until the data set is not significantly improved by further exclusions. The exclusion can be performed on whole sweeps, or sets of image groups within sweeps, to enable only the end of sweeps to be removed in the case of radiation damage. The scaling and exclusion uses successive cycles of the scaling-optimization algorithm, without using full-matrix minimization, and the ΔCC_1/2_ algorithm, followed by a final cycle of the scaling-optimization algorithm with full-matrix minimization. A number of termination criteria may be used, including the number of cycles, the data-set completeness and the percentage of observations removed. The usage of this option is intended as a semi-automated tool, enabling an exploration of the evolving characteristics of the scaled data set as the most non-isomorphous data are successively removed.

### 3.6. Free-set validation of scaling models   

An important consideration for scaling and merging is the suitability of a particular choice of model parameterization; however, precision indicators such as *R*_p.i.m._ and CC_1/2_ can be misleading, particularly if the model is minimized against a subset of the data. Increasing the number of model parameters will generally increase the precision of the data by improving the agreement between symmetry-related reflections, yet this may result in overfitting experimental noise rather than modelling a true signal in the data. To assess the impact of model or algorithm choices on the scaling-model quality, a free-set validation option has been implemented, analogous to the free-set validation used in macromolecular refinement (Brünger, 1992 ▸, 1993 ▸). It is important to stress that the free-set validation does not form part of the recommended routine scaling in *DIALS*, but exists as an additional tool.

In the general method of free-set validation, the data set is split into a work and a free set, with the free set typically containing 5–10% of the data. The model (for example atomic coordinates) is minimized using only the work set, and by testing the fit of the model to the free set using a suitable metric one can obtain additional insights into the suitability of the refined model. When comparing models, an improved model should improve the precision of data in both the free and work set, although it must be stressed that an increase in *precision* does not necessarily lead to a more *accurate* data set. In scaling, the purpose is to increase the precision to the extent allowed by the quality of the data, but no further. The selecting and testing of free sets can be repeated with different subsections of the data to increase the significance of the results, *i.e.* *n*-fold validation, to avoid large fluctuations based on the particular free set chosen (Brünger, 1993 ▸).

In the implementation for scaling within *DIALS*, the free set is chosen by taking a percentage of the groups of symmetry-equivalent reflections, typically 10%, so that all symmetry equivalents of a given reflection are fully contained in either the work or the free set. Only the work set is used to determine parameter values during the scaling algorithm, with a subset of the work-set reflections typically used in the minimization procedures. The refined models are then applied to both the work-set and free-set reflections, as well as outlier rejection being performed on both sets. This algorithm can be repeated *n* times until all of the data have been used as part of a free set once, with the resulting metrics being averaged over the repeats. *R*_meas_ and CC_1/2_ metrics are reported for the work and free set, with the *R*_meas_ metric being favoured over *R*_p.i.m._ owing to the fact that it is independent of multiplicity, as the multiplicities of the work and free set are in general unequal. Typical uses for this approach in scaling would be to investigate the effect of including particular model components, changing the parameterization for the individual model components or assessing the effects of algorithm choices. Furthermore, a large discrepancy between the work and free metrics is a simple indicator of deficiencies in the current model.

## 4. Scaling of example data sets   

To validate the new scaling algorithms within *DIALS* and to demonstrate the use of the tools, the results of scaling a variety of experimental data sets are shown in this section, including a weak macromolecular data set, a multi-crystal narrow-sweep data set with significant radiation damage and a multi-crystal data set. The scaling algorithms in *DIALS* can be run using the command-line program *dials.scale*, with optional keyword parameters. The full commands used to analyse each example data set are given in Appendix *B* [App appb], and the majority of plots shown can be found in the HTML report generated by *dials.scale*.

### 4.1. Scaling of high-multiplicity weak data   

**Figure 5:**
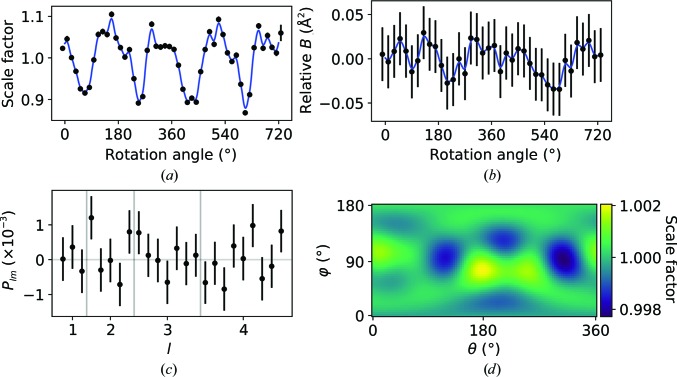
Scaling-model components determined for the weak but high-multiplicity thermolysin data set. The error bars indicate the standard parameter uncertainties determined from the final minimization cycle. (*a*) Inverse scale-factor correction for the scale correction *C_hl_* (parameter uncertainties are too small to distinguish). (*b*) Smoothly varying *B* factor for the decay correction *T_hl_*. (*c*) Values of the spherical harmonic coefficients *P_lm_* that define the absorption surface correction. (*d*) The angular dependence of the absorption surface correction factor *S_hl_* in the crystal frame (*i.e.* *S_hl_* plotted for −**s**_0_ = **s**_1_).

**Figure 6:**
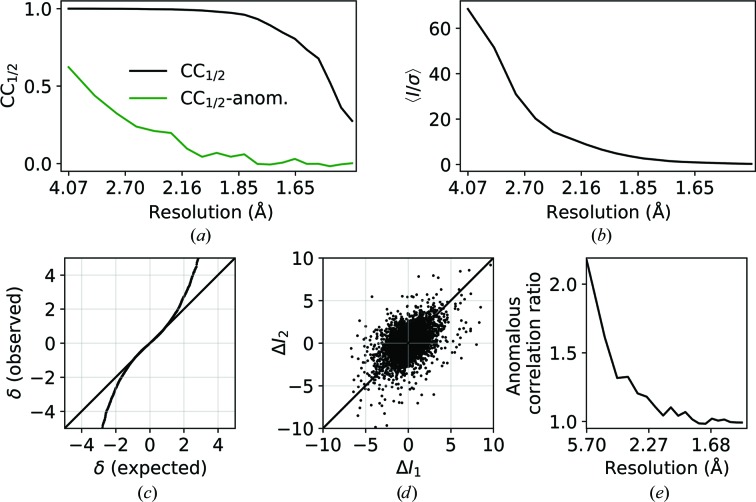
Resolution-dependent CC_1/2_ (*a*) and 〈*I*/σ〉 (*b*) for the scaled data set. (*c*) Normal probability plot of anomalous differences δ_anom_ = (*I* ^+^ − *I* ^−^)/[σ^2^(*I* ^+^) + σ^2^(*I* ^−^)]^1/2^ (*d* > 2.13 Å). (*d*) Scatter plot of Δ*I*_anom_ pairs (Δ*I*_1_ = 〈*I* ^+^〉_1_ − 〈*I* ^−^〉_1_, Δ*I*_2_ = 〈*I* ^+^〉_2_ − 〈*I* ^−^〉_2_ for random splitting of *I* ^+^ and *I* ^−^) and (*e*) anomalous correlation ratio for acentric reflections [ratio of r.m.s. deviation along *y* = *x* to r.m.s. deviation along *y* = −*x* in (*d*)].

To test the handling of data for macromolecular crystallo­graphy, a weak high-multiplicity data set from a thermolysin crystal was used. Diffraction data were collected on beamline I03 at Diamond Light Source, consisting of 7200 images at 0.1° intervals, following a low-dose high-multiplicity strategy (Winter & McAuley, 2016 ▸). The integrated data were processed with *dials.scale* and a summary of the merging statistics is shown in Table 1 ▸. Plots of the scaling-model components are shown in Fig. 5 ▸, and resolution-dependent statistics and plots of the anomalous signal are shown in Fig. 6 ▸.

The 180° periodicity is evident in the scale component, and some features are evident in the absorption correction surface, although the relative variation in this correction is well below 1%. The relative *B* factor shows no major deviation from its null value, as expected given the low-dose collection strategy. Despite the fact that 〈*I*/σ(*I*)〉 tends towards zero at high resolution, a significant half-data set correlation is maintained to the highest resolution, with significant anomalous correlation for *d* > 2.13 Å (significant at *p* = 0.01). Furthermore, the anomalous signal is evident in the anomalous difference plots (Fig. 6 ▸).

**Figure 7:**
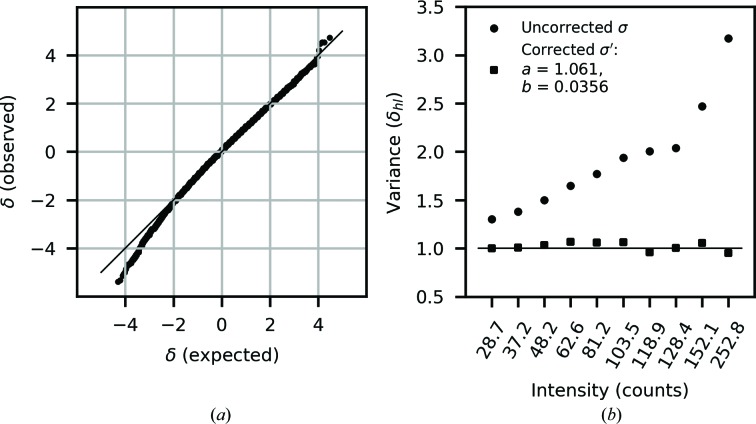
(*a*) Normal probability plot of the normalized deviations δ_*hl*_ after error-model correction compared with an expected normal distribution (solid line), showing good overall agreement but with some discrepancy for deviations below −2. (*b*) Comparison of the variance of the normalized deviations, binned by intensity, before and after error-model correction, which reduces the variances close to the target of unity across the intensity range.

Error-model optimization determined parameters of *a* = 1.061 and *b* = 0.036, suggesting an *I*/σ asymptotic limit of 26.5. The normal probability plot of δ_*hl*_ and the intensity-binned variances, for the data used for error-model optimization, are shown in Fig. 7 ▸. The normal probability plot shows good agreement with a normal distribution, but with some deviation at greater than two standard deviations from the mean. The intensity-binned variance plot shows that the error-model correction is able to bring the variance of δ_*hl*_ into much closer agreement with the expected value of unity across the intensity range, with the greatest effect at high intensity owing to the error-model *b* term.

To confirm the suitability of the scaled data set, phasing was performed by molecular replacement using *Phaser* (McCoy *et al.*, 2007 ▸) with PDB entry 2tlx (English *et al.*, 1999 ▸) as the search model (without water or ligands), resulting in a TFZ score of 15.2, an LLG of 260 and a refined LLG of 8029. The structure was refined using *REFMAC*5 (Murshudov *et al.*, 2011 ▸) using a free set of 2413 reflections (5%). The final *R*-value statistics obtained were *R*_work_ = 19.1% and *R*_free_ = 21.6% for a model including Zn^2+^, Cl^−^, Ca^2+^ and SO_4_^2−^ ions and a short peptide.

To confirm the suitability of the scaling model and evaluate the effect of including the approximately null decay correction, a free-set validation comparison was performed. The work/free-set values were practically identical; including the decay correction gave an overall work and free *R*_meas_ of 0.21759 and 0.21959, respectively, compared with values of 0.21760 and 0.21956 without the decay correction, and the overall CC_1/2_ values were identical for each case. The low gap between the free-set and work-set statistics indicates negligible over­parameterization, as expected for a high-multiplicity data set, while the free-set statistics show that the inclusion of the ‘null’ decay component has no implication for the scaled data set.

### 4.2. Scaling multi-crystal *in situ* data with* DIALS*   

To demonstrate the new approaches available for scaling multi-crystal data in *DIALS*, previously published *in situ* data for *Haemophilus influenzae* TehA (TehA; Axford *et al.*, 2015 ▸) were investigated. The data set investigated consisted of 72 sweeps with oscillation ranges of 4–10°. The data were integrated using *xia*2 (Winter, 2010 ▸) and a symmetry analysis was performed using *dials.cosym*, which determined the Laue group to be *R* 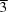, resulting in an assigned space group of *R*3:H, and reindexed all sweeps to a consistent setting.

**Figure 8:**
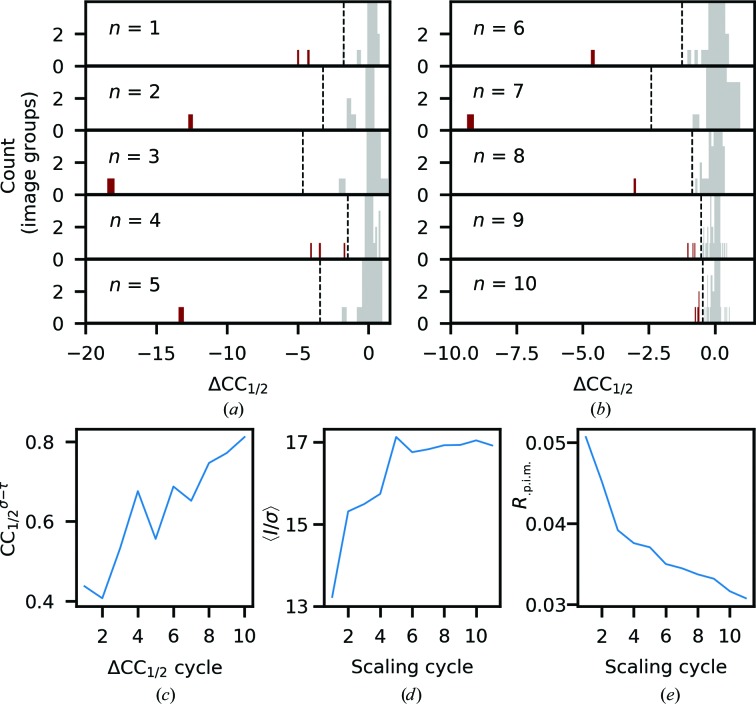
Initial exploratory scaling and filtering analysis on a multi-crystal TehA data set on 263 groups of ten images. (*a*) The distribution of ΔCC_1/2_ values for the image groups after each round (*n*) of scaling, with the plots limited to counts of 4 and below to display low-count histogram bins. Groups with ΔCC^*i*^_1/2_ < 〈ΔCC_1/2_〉 − 4σ are shown in red and were removed by the filtering algorithm (the 4σ cutoff is indicated by the dashed line). From the ninth cycle, the lowest ΔCC^*i*^_1/2_ could be considered to be within the tail of the central distribution. (*b*, *c*, *d*) Resolution-averaged CC_1/2_^σ–τ^, 〈*I*/σ〉 and *R*_p.i.m._ per cycle, which show significant improvement in the first seven cycles of scaling and filtering, with more gradual improvement towards the end of the ten cycles.

Given the potential for non-isomorphism and radiation damage, an exploratory analysis was performed using ten cycles of scaling and ΔCC_1/2_ exclusion. A *d*_min_ cutoff of 2.3 Å was applied, while the ΔCC_1/2_ analysis was performed on groups of ten images to allow exclusion of only the end of sweeps to account for systematic errors owing to radiation damage. Image groups were excluded if their ΔCC_1/2_ values were 4σ below the mean value. The results of this scaling and filtering are shown in Fig. 8 ▸.

**Figure 9:**
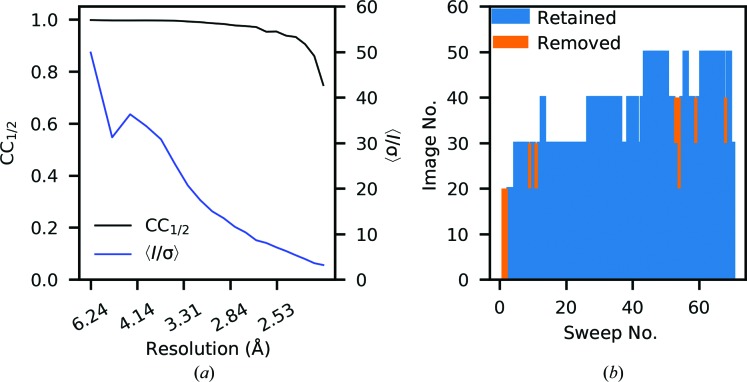
(*a*) CC_1/2_ and 〈*I*/σ〉 of the selected data set (after eight cycles of scaling and exclusion). (*b*) The image ranges retained or removed for the selected data set. The whole of the first two sweeps were removed, in addition to the ends of several other sweeps, removing 3.4% of the reflections from the initial data set.

Initially, significant improvements were made in the data quality, as the most systematically different data are removed. As can be seen from the histogram plots of ΔCC_1/2_, from the ninth filtering cycle onwards the extremal ΔCC_1/2_ values are significantly reduced, and the merging statistics show more gradual improvement towards the end of the ten cycles of scaling and filtering. In the last two filtering cycles, the analysis is starting to remove some groups which could be considered to be in the tail of the central distribution of ΔCC_1/2_ values. Based on these insights, the initial data were rescaled, excluding all regions identified by the first eight filtering cycles (this excludes 3.4% of the observations). Plots of the data-set statistics, as well as a plot of the retained/excluded image ranges, are shown in Fig. 9 ▸, while the data-set statistics, including refinement, are shown in Table 1 ▸. While further improvement could be obtained by further exclusion, in this example a conservative approach is taken. It is for this reason that this tool is described as semi-automated, as some user discretion is required to make a decision on when to stop excluding data. It is not clear that there is a suitable metric for an appropriate automatic cutoff that would be applicable to all data sets, as the form of the distribution of ΔCC_1/2_ values is not well characterized. In this example, a cutoff of 4σ was used; however, other cutoffs could be equally valid for exploratory purposes, which may result in a slightly different, although functionally similar, set of data for the final data set. In the example, the decision on the final data set is guided by the observation that the majority of the improvement appears to have been achieved by the end of the eighth cycle.

The data set after eight cycles of scaling and filtering was used for structure determination to verify the scaled data set. The scaled data were merged and truncated with *dials.merge* and molecular replacement was performed using *Phaser* with the search model PDB entry 4ycr (Axford *et al.*, 2015 ▸) with no water or ligands. The structure was refined with *REFMAC*5, adding waters but without building ligands into the model. The final *R*-value statistics obtained were *R*_work_ = 15.1% and *R*_free_ = 18.6%, verifying the quality of the subset of data determined in this manner. In comparison, the previous published analysis used data from 56 crystals, consisting of 99 220 reflections to a resolution limit of 2.3 Å, to determine a structure with *R*_work_ = 15.6% and *R*_free_ = 20.0% (Axford *et al.*, 2015 ▸). Although the results of this analysis are not directly comparable to the previously published analysis, as a different subset of the data are used, as well as different data-analysis programs, we note that slightly more favourable *R* factors are obtained, confirming the validity of this approach. The analysis presented here suggests that, as judged from the merging statistics, a scaling/ΔCC_1/2_ analysis can in fact identify a small number of image groups that are the most systematically different parts of the data. In general, in the analysis of such data sets, a compromise must be made between obtaining a data set with higher completeness and multiplicity and obtaining a data set with reduced radiation damage. The tool presented here allows the retention of completeness and multiplicity, providing a filtered data set that can be used as a starting point for further assessments of radiation damage.

### 4.3. Incremental scaling to build a merged data set   

**Figure 10:**
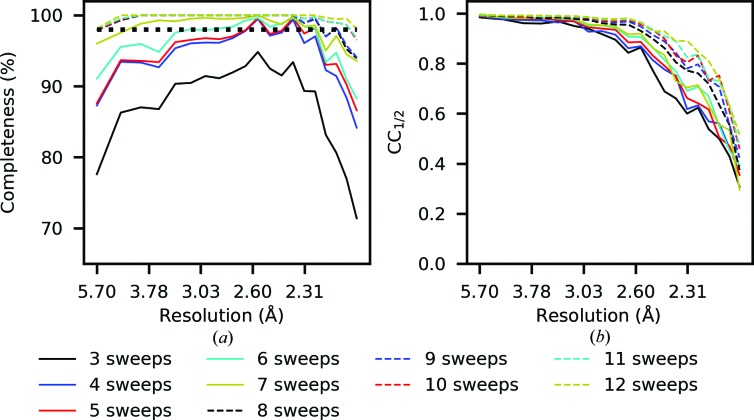
Incremental scaling of 20° rotation sweeps collected *in situ*. (*a*) Completeness and (*b*) correlation coefficient for the number of sweeps in the combined data set. Each sweep is added individually to the combined scaled data set measured up to that point, triggering the reference-scaling algorithm. As each sweep is added, the completeness is monitored until a given completeness is obtained across the resolution range (98% in this example, which is obtained after adding sweep 12).

As discussed in Section 3.4, *dials.scale* also supports an incremental workflow for scaling multi-sweep/multi-crystal data sets, which can be used to support recent new data-collection strategies (Winter *et al.*, 2019 ▸). This functionality could be used to guide data-collection strategies, particularly for *in situ* experiments, where measurements from many crystals need to be combined until a data set with the desired characteristics is obtained. This can be demonstrated with the following example, using data collected on the VMXi beamline at Diamond Light Source (Sanchez-Weatherby *et al.*, 2019 ▸) consisting of 20° rotation measurements on crystals of thaumatin. Following data integration with *xia*2 using *DIALS*, the first three data sets were scaled together: this suggests a resolution limit of 2.1 Å based on a CC_1/2_ threshold of 0.3; however, the completeness is insufficient. It is important to note that the resolution on VMXi was also limited by the detector size. Overall, the completeness is 87.4% for a resolution limit of 2.1 Å; however, it falls below 80% in both the lowest and the highest resolution shells. Based on this initial assessment, a typical experimental strategy would be to continue collecting until reaching a given completeness. In this demonstration, a target completeness of 98% in all resolution shells was chosen. Each subsequent integrated data set was added individually to the combined scaled data set, triggering the reference-scaling algorithm (Fig. 3 ▸) and restoring the scaling models for the already scaled sweep to aid in convergence of the scaling algorithm. After the 12th data set was added, a completeness of 98% was obtained across the resolution range. The resolution-dependent completeness and CC_1/2_ per scaling run are shown in Fig. 10 ▸.

While this analysis was performed using repeated runs of the command-line program, one could integrate such functionality into a server-type application for more efficient algorithm execution by retaining the data set in memory and bypassing some of the initial setup of data structures. In this example use case non-isomorphism was not considered, which could be a significant issue for certain systems. To enhance this approach, one could use workflows involving periodic non-isomorphism investigation using a ΔCC_1/2_ analysis or intensity clustering; however, it may be preferable to use the approach outlined in Section 4.2, depending on the nature of the experiment.

### 4.4. Validation of the reflection-selection algorithm   

**Figure 11:**
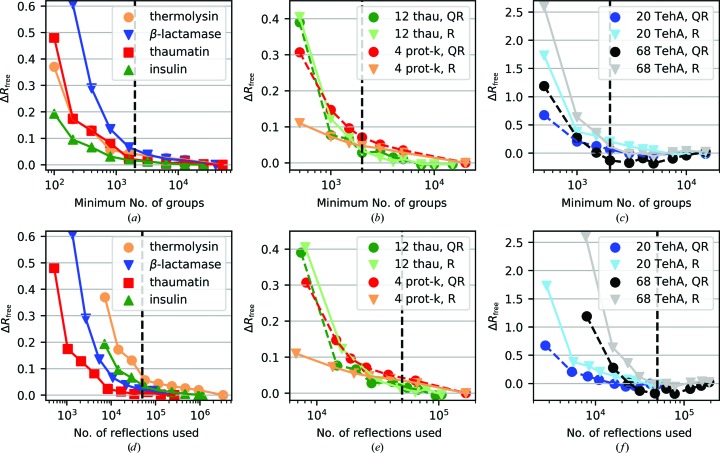
The difference in the free *R*_meas_ determined using all reflections and using a subset of reflections [Δ*R*_free_ = free *R*_meas_ − free *R*_meas_(all)] plotted against the minimum number of groups (top) and the number of reflections in the subset (bottom) for (*a*, *d*) single-sweep data sets, (*b*, *e*) multiple-sweep data sets and (*c*, *f*) a multi-crystal data set. For single sweeps a random group selection is used, whereas for multiple-sweep and multi-crystal data a random (R) and a quasi-random (QR) algorithm are tested. Based on the observed trends, two criteria were chosen for the reflection subset to be used for scaling-model optimization: it must contain at least 2000 groups and 50 000 reflections, as indicated by the vertical dashed lines.

Motivated by the desire to use as few reflections as necessary during scaling-model optimization to improve computational efficiency, without sacrificing the quality of the scaling result compared with using all reflections, a free-set validation analysis was performed to determine suitable parameters for the reflection-selection algorithms. The effect of changing the number of randomly selected groups of symmetry equivalents was investigated, as well as the difference between the random and quasi-random algorithms for multi-sweep data sets, the results of which are shown in Fig. 11 ▸.

For single-sweep scaling, four data sets were investigated; two high-multiplicity data sets of thermolysin (investigated in Section 4.1; multiplicity *m* = 73.2) and insulin (*m* = 71.4), and two lower multiplicity data sets of thaumatin (*m* = 4.6) and β-lactamase (*m* = 6.7). For the high-multiplicity data sets, using as few as 800 groups of symmetry equivalents results in an almost identical free *R*_meas_ compared with using all reflections, *i.e.* a Δ*R*_free_ ≲ 0.05%, whereas for the lower multiplicity β-lactamase data set a much higher number of groups is required. Considering the trends as a function of total reflections used, shown in Fig. 11 ▸(*d*), the lower multiplicity data sets show a lower Δ*R*_free_ for a given number of reflections, with around 20 000 reflections needed for the lower multiplicity data sets to have a Δ*R*_free_ of ≲0.05%. Overall, this means that choosing a single threshold based on the number of reflections/groups is unsuitable, as one must also consider that a minimum number of groups/reflections should be used for high- or low-multiplicity data sets, respectively.

For multi-sweep scaling, three data sets were investigated: the multi-crystal TehA data set investigated in Section 4.2, a 12-sweep thaumatin data set (Section 4.3) and a four-sweep proteinase K data set (Sandy *et al.*, 2019 ▸). For the TehA data set, the validation was performed on the 68 sweeps resulting from the scaling and filtering, and also on a subset of 20 complete sweeps (sweeps 12–31) for comparison. For both data sets, the quasi-random reflection selection results in a significantly improved result for a given number of groups/reflections, particularly for a low number of groups, while for the thaumatin data set both algorithms give similar results. For the proteinase K data set, the quasi-random selection gives an inferior result when fewer than 2000 groups are used: this was owing to the fact that for this data set the well connected groups selected contained an uneven distribution of reflections across data sets (for example, for 500 groups the subset contained only 10% of one sweep yet 37% of another). Overall, these results indicate that the quasi-random algorithm is preferable for scaling a large number of sweeps, as in the case of multi-crystal data, as a more suitably connected reflection subset is chosen compared with a random selection. For wider sweeps, the selection algorithms are comparable if using a sufficient number of groups to ensure an approximately uniform distribution of reflections across data sets.

Based on these results, the following defaults were chosen for *dials.scale*, although the algorithm choice and parameters remain under user control, as detailed in Appendix *B* [App appb]. For both single-sweep and multi-sweep data, two criteria must be met in selecting the reflection subset for model optimization: it must contain at least 2000 groups of symmetry equivalents and at least 50 000 reflections; these threshold values are indicated by the vertical lines in Fig. 11 ▸. These values would place the example data sets well within 0.1% of the free *R*_meas_(all) values, *i.e.* a functionally equivalent scaling result, and ensure a sufficient number of reflections for both the low-and high-multiplicity data sets. With these thresholds in place sufficient sampling is obtained for cases such as the proteinase K data set, and therefore the quasi-random algorithm can be used by default for multi-sweep scaling. It is anticipated that this will have the greatest benefits when scaling a large number of sweeps by requiring only a low fraction of reflections for minimization, or in cases where the per-sweep multiplicity is low by helping to choose reflection groups that best constrain the model optimization.

The advantage of this approach, as opposed to using a fixed percentage of the data, is to limit the data needed for large data sets to allow practical scaling while ensuring optimal use of the data for lower multiplicities, whether the low multiplicity is owing to low symmetry or owing to a narrow measurement sweep. For example, for the high-multiplicity thermolysin data set the default thresholds use 4% of the observations. As the scaling-model refinement scales approximately linearly with the number of reflections used, the total scaling runtime is reduced by over 50% when using 4% of the observations compared with using 30%. Given that the number of parameters used for full-rotation data is typically below 100, the thresholds ensure an observation-to-parameter ratio of over 500 for single-sweep data, while for the TehA data set the observation-to-parameter ratio is still high at approximately 100. The low Δ*R*_meas_ values of the free-set validation confirm that for this order of observation-to-parameter ratio the general risk of scaling-model overfitting is very low. The insights from this validation analysis can also be used by automated processing pipelines, such as *xia*2, to adjust the algorithm choice and thresholds based on the properties of the input data.

## 5. Conclusions   

Scaling algorithms have been implemented in *dials.scale*, which is part of the *DIALS* software package, incorporating well established scaling approaches and introducing specialized tools for multi-crystal data scaling, validation of scaling parameterization and optimization approaches, and a high degree of flexibility to its workflow. The effectiveness of the implementation has been demonstrated on example macromolecular crystallographic data.

It was demonstrated how the validation tool can be used to instruct the implementation of an algorithm component, namely reflection selection, enabling a methodical approach to future algorithm development and testing. The analysis presented confirmed that using a subset of reflections for scaling-model optimization gives a functionally equivalent result to using all reflections, which need only be a small fraction of the total observations for high-multiplicity data sets. Although the default *physical* model parameterization is appropriate for many use cases, with a low risk of overfitting the data, the free-set validation tool can guide a user in the construction of a tailored scaling model for more complex and challenging data sets, allowing the impact of overfitting to be quantified. For multi-crystal scaling, a scaling and exclusion tool was presented which provided a simple, automated way to explore the evolution of a *H. influenzae* TehA data set as the least isomorphous data were removed, giving a substantial improvement in merging statistics while only removing a small fraction of the data. The scaling workflows described for handling multi-sweep data sets, in addition to other tools available in *DIALS*, are examples of how the package can be used to provide a high degree of flexibility in the design of workflows and the approaches taken. The development of the algorithms presented have benefitted from extensive testing on experimental data measured at Diamond Light Source beamlines: data reduction using *DIALS* tools has been implemented as the default workflow for *xia*2 since the release of *DIALS* 2.0.

In summary, the implementation of symmetry-determination and scaling algorithms in *DIALS* extends the capabilities of the package, allowing processing from raw diffraction images to scaled, merged data suitable for structure determination using *DIALS*. It is intended that the tools described here will enable the investigation and implementation of new algorithms and approaches to post-integration data reduction, furthering our ability to deal with increasingly inventive ways of measuring macromolecular crystallography data. 

## Figures and Tables

**Table 1:** Crystallographic parameters, data-set statistics and renement statistics for data scaled with *DIALS* and solved by molecular replacement and renement (see text) Values in parentheses are for the outer shell.

	Thermolysin	TehA
Crystal parameters		
Space group	*P*6_1_22	*R*3:H
Unit-cell parameters (Å)	*a* = *b* = 92.339, *c* = 127.707	*a* = *b* = 98.434, *c* = 136.012
Data-set statistics		
Resolution (Å)	79.97–1.50 (1.53–1.50)	72.23–2.30 (2.34–2.30)
CC_1/2_	1.000 (0.262)	0.998 (0.748)
*R*_merge_	0.216 (5.965)	0.135 (0.745)
*R*_meas_	0.217 (6.025)	0.140 (0.780)
*R*_p.i.m._	0.025 (0.811)	0.032 (0.224)
〈*I*/σ(*I*)〉	12.3 (0.2)	16.9 (3.2)
Completeness (%)	97.58 (83.09)	95.23 (93.62)
Multiplicity	72.9 (51.1)	14.3 (9.9)
Observations	3700696 (108764)	297618 (10428)
Unique reflections	50774 (2128)	20821 (1057)
Wilson plot *B* factor (Å^2^)	20.2	48.5
Refinement statistics		
Work-set reflections	45981	19824
Free-set reflections	2413	994
*R*_work_ (%)	19.1	15.1
*R*_free_ (%)	21.6	18.6

## Appendix A Implementation details of error-model optimization

The error model in *DIALS* is a two-component model, with the updated form of the variance given by

The error model is optimized by assessing the distribution of normalized deviations δ_*hl*_ under the assumption that the expected distribution is normal. This assumption only holds when the Poisson distribution owing to counting statistics can be sufficiently described by a normal distribution; therefore, the model is only minimized on reflection groups with 〈*I_h_*〉 > 25.0. Furthermore, if a significant contribution to the variance is uncertainty in the background, then the assumption may not hold; therefore, only reflection groups with 〈*I*/σ^2^〉 > 0.85 are used.

To determine the *a* parameter, a linear fit is made to the central region (|*x*| < 1.5) of the normal distribution plot of δ_*hl*_ for a given *b*, with the fitted slope used as the updated estimate for *a* (*i.e.* the slope of the normal probability plot would be 1 after correcting with this value of *a*). To determine the *b* parameter, the data are divided into logarithmically spaced intensity bins. A minimization is performed, with a fixed *a*, using an adaptation of a previously described target function (Evans & Murshudov, 2013 ▸),

where *v_i_* is the variance of the δ_*hl*_ distribution in intensity bin *i* and *w_i_* is the bin weight. The addition of a 1/*v* term reflects the mathematical constraint that a variance below zero is impossible, and a variance close to zero should be strongly avoided for a good model. The modification to the square term is chosen such that the target function has the minimum at *v* = 1. A weight proportional to 〈*I*〉 is used to favour obtaining a good agreement for the stronger reflections, which are most affected by the correction. The *a* and *b* parameters are sequentially estimated in this manner until convergence is achieved.

## Appendix B Program usage within the *DIALS* framework

As with other programs in the *DIALS* framework, the scaling program can be run from the command line by providing integrated reflection data and model information. The command used to analyse the thermolysin data set in Section 4.1 is as follows:

To validate the scaling-model decay correction, the following command was used:

The exploratory multi-crystal analysis in Section 4.2 was performed with the following command (using the output data files from *dials.cosym*):

After deciding on the appropriate subset of the data, the data set was scaled using the command

The incremental scaling analysis in Section 4.3 was performed with the following commands. To scale the first three sweeps

and thereafter each individual sweep was added using a command such as

To perform the validation analysis in Section 4.4, additional options of the following form were used:

To vary the reflection-selection parameters when not performing validation, the relevant parameters are
